# Observation of the therapeutic effect of auricular bean pressing on early knee osteoarthritis pain: A randomized controlled trial

**DOI:** 10.3233/BMR-220271

**Published:** 2023-06-30

**Authors:** Yeyan Lin, Yongqin Wu, Xuelai Zhou, Bin Shen, Cunxian Lv

**Affiliations:** a Department of Orthopaedics, Wenzhou TCM Hospital of Zhejiang Chinese Medical University, Zhejiang, China; b School of Nursing, Wenzhou Medical University, Wenzhou, Zhejiang, China

**Keywords:** Auricular bean pressing, knee osteoarthritis, pain, curative effect

## Abstract

**BACKGROUND:**

In the treatment of knee osteoarthritis (KOA), there is a need for the long-term use of therapeutic drugs that reduce joint pain and have fewer adverse effects.

**OBJECTIVE:**

This study aimed to investigate the therapeutic effect of bean pressing on ear points on early KOA pain.

**METHODS:**

One hundred patients with KOA recruited at the Wenzhou Hospital of Traditional Chinese Medicine between February 2019 and May 2022 were divided randomly into a treatment group (n= 50) and control group (n= 50). Patients in the treatment group received regular rehabilitation combined with auricular bean-pressing treatment, while patients in the control group only received conventional rehabilitation treatment. The measurement indicators – knee swelling, tenderness, range of motion sign score, C-reactive protein, and the Western Ontario and McMaster Universities Osteoarthritis (WOMAC) indexes – were recorded before and after treatment.

**RESULTS:**

On day 5 following the start of treatment, the visual analog scale (VAS) and WOMAC scores of the treatment group were significantly lower than those of the control group (P< 0.05), and the VAS and WOMAC scores in the treatment group after treatment were significantly lower than those before treatment (P< 0.05). At week 4 after the start of treatment, the dosage of nonsteroidal anti-inflammatory drugs (NSAIDs) in the treatment group was significantly lower than that in the control group (P < 0.05). No adverse events were observed during the treatment.

**CONCLUSIONS:**

Auricular bean-pressing therapy had an analgesic effect and could also alleviate mild to moderate KOA swelling, joint stiffness, and other symptoms, effectively reducing the demand for NSAIDs and improving both knee function and quality of life. The results suggested that auricular bean-pressing therapy has promising prospects in the treatment of early KOA pain.

## 1. Introduction

Knee osteoarthritis (KOA) is pathologically characterized by cartilage destruction, degeneration, bone hyperplasia, aseptic synovitis, and ligament degeneration. Its clinical manifestations include pain, swelling, and limited mobility, and in severe cases, it may lead to knee disability, which more frequently occurs in middle-aged and elderly people [1]. The main goal of treating KOA is to reduce pain and improve function [2]. Several options are available to treat KOA, including weight loss, lifestyle advice, exercise therapy, nonsteroidal anti-inflammatory drugs (NSAIDs), corticosteroid injections, and glucosamine supplements. The most commonly used pain treatment drugs are NSAIDs, but their long-term use increases the risk of gastrointestinal bleeding and vascular adverse events [3]. Therefore, there is a need for the long-term use of therapeutic drugs that reduce joint pain and have fewer adverse effects.

Auricular point bean pressing involves pressing and fixing cowherb seed on the auricular points to continuously stimulate them and create a therapeutic effect [4]. The method of pressing auricular points to prevent and treat diseases as a treatment method in China has a history of over 2,000 years. In recent decades, its application has increased considerably, particularly for conditions characterized by pain. According to previous literature [5, 6], auricular point bean pressing (on knee acupoints, for example) has a beneficial effect on relieving pain in patients with KOA who have been treated conservatively or surgically. Other acupoints, such as the adrenal, subcortical, and endocrinal acupoints, are closely associated with sedative and analgesic effects, which are beneficial for relieving the symptoms of KOA. However, there are few studies on the application of auricular point bean pressing to treat early KOA. The present study intervened in early KOA cases using auricular point bean pressing and studied its effect on the Western Ontario and McMaster Universities Osteoarthritis (WOMAC) indexes of function, pain, and stiffness to determine whether acupressure on four pre-selected specific auricular points could relieve joint pain, reduce NSAID consumption, relieve symptoms, and improve functioning. The results provide an evidence-based foundation for clinical treatment.

## 2. Materials and methods

### 2.1 Clinical data

This was a randomized controlled trial. One hundred participants were selected from patients with KOA who were treated at the Department of Orthopedics of Wenzhou Hospital of Traditional Chinese Medicine between February 2019 and May 2022. The standard follow-up for all treated patients lasted 4 weeks. The study was approved by the ethics committee of the Wenzhou Hospital of Traditional Chinese Medicine Affiliated to Zhejiang University of Traditional Chinese Medicine (approval no. WTCM-H-KT-2019047), and all patients provided signed informed consent for their inclusion in the research. The study met the requirements of the Declaration of Helsinki.

One hundred patients with KOA enrolled in this study were divided into control and study groups (n= 50 each), respectively, using a random number table method. The patients in the treatment group received routine rehabilitation combined with auricular point bean-pressing treatment, while the patients in the control group received routine rehabilitation treatment. 

The inclusion criteria were: (1) patients > 40 years old and (2) patients with early KOA with a Kellgren–Lawrence (KL) [7] image grading of Grade I or II. Radiological evaluation of the patients was performed by two experienced observers who had worked in the orthopedic field for many years.

The exclusion criteria were: (1) patients who had undergone knee surgery, (2) patients who had undergone intra-articular injection treatment within 3 months before the study treatment, (3) patients who could not complete the data collection form, and (4) patients with ear eczema or a history of any ear surgery.

### 2.2 Therapeutic methods

**Figure 1.**
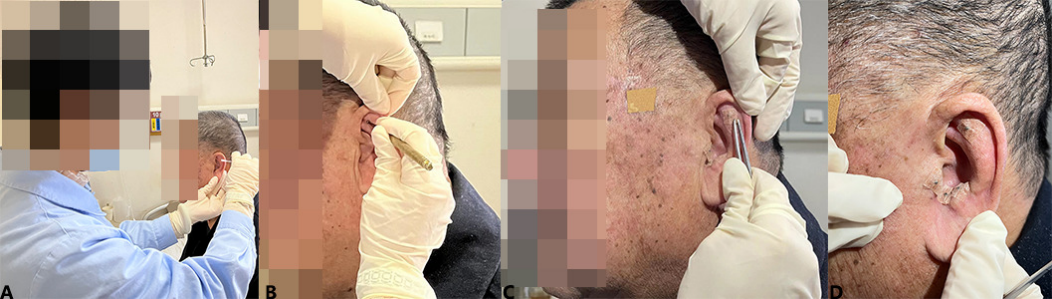
Auricular bean-pressing process. (A) Disinfection; (B) Selected auricular acupoints; (C) Fixed Cowherb seeds: (D) Finished.

The control group received routine rehabilitation treatment. In both groups, the patients required NSAIDs during treatment and follow-up; this was prescribed as oral celecoxib (Pfizer Inc., USA) as an anti-inflammatory and for pain relief, one tablet twice a day. Functional exercises were prescribed: isometric training, e.g., straight leg raising and plantar dorsiflexion as the main methods for enhancing muscle strength when the pain was severe or in the acute stage. Joint loosening training included femoral-tibial and proximal tibiofibular joint long-axis traction, patellofemoral lateral and up-down sliding, patellofemoral joint separation, lateral horizontal pushing of the femorotibial joint, patella sliding, and knee extension swings [8]. Each of the above movements was held for 30 s, and 30 training exercises were completed each day. Following pain relief, resistance training for the quadriceps and other muscles around the knee was performed. The patient’s legs were fixed, and the knees could sag naturally; then, the affected limbs were actively extended, and the knees were flexed 50 times/d [9].

The treatment group received auricular point bean-pressing therapy based on the rehabilitation treatment. For auricular disinfection, a cotton swab was pressed onto the acupoints (Fig. 1A). Four auricular acupoints on the same side as the affected knee were selected; these corresponded to the knee and to adrenal, subcortical, and endocrinal [10] functions (Fig. 1B). Cowherb seeds (Tai Cheng Company) were fixed and pressed in an alternative press–release pattern, moving from light to heavy pressure (Fig. 1C). Each acupoint was pressed 20 times, for 5 s each time, to strengthen the stimulation. The pressing strength was sufficient for turning the auricle red, warm, or sore while remaining tolerable. The acupoints on both ears were pressed alternately for 3 min/acupoint, 3 times/d. The cowherb seeds were changed every 3 d, and the course of treatment was 4 weeks [11] (Fig. 1D). The cowherb seeds were fixed to the side where the KOA was observed and were pressed daily in the morning, at noon, and in the evening, with each time lasting approximately 3 min. Pressing was stopped when the patient experienced soreness, numbness, or fever. Patients received the guidance of professional traditional Chinese medicine (TCM) clinical staff to maintain their compliance and ensure accurate acupoint pressure performance.

### 2.3 Treatment indexes

To assess the efficacy of treatment on knee function and pain, the WOMAC index scores [12], the pain visual analog scale (VAS) [13], and serum C-reactive protein (CRP) before and 1 month after treatment were employed as the primary outcomes to evaluate patients. Additionally, the patients’ dependence on celecoxib was a secondary outcome in this study.

The WOMAC is used mainly to evaluate the degree of KOA in patients. The scale includes three dimensions: pain, stiffness, and joint function, with 24 evaluation items. A five-grade scoring method was adopted, and each item was scored from 0 to 4 points according to severity (maximum score: 96 points). The higher the score, the more serious the condition.

The VAS scale comprised a 100-mm-long line with anchoring descriptors. The patient marked the line to indicate their pain perception, and the result was expressed as the distance from the left end point to the marked point (in mm). The maximum score was 10 points. The lower the score, the better the pain relief effect.

The CRP level with high sensitivity can provide effective information for many types of chronic diseases, and its value greatly increases in the early stage of inflammation. In the present study, 5 mL of fasting venous blood was collected from the patient in the morning and centrifuged at high speed. Serum was obtained, and the CRP value was detected by radioimmunoassay.

### 2.4 Statistical analysis

We used SPSS™ Statistics v25.0 software (IBM, Chicago, USA) for all data processing. The data with normal or approximately normal distribution were expressed as mean ± standard deviation ($\bar{x}$± sd). An independent-samples t test was performed to compare differences between groups, while intra-group differences were evaluated by a paired t test. Count data were expressed as case percentages (%) and compared between groups using the Chi-squared ($\chi^{2}$) test. The inspection level was set at α= 0.05, and P< 0.05 was considered statistically significant.

## 3. Results

### 3.1 General information statistics

**Table 1:** Comparison of general data of patients between two groups

Group	Treatment group	Control group	P value
Number of cases	50	50	
Gender (male/female)	21/29	23/27	0.687
Age	69.30 ± 5.06	67.24 ± 5.34	0.050
WOMAC total score	71.30 ± 5.19	70.00 ± 4.43	0.181
Kellgren grade (number) (II/III)	23/27	24/26	0.841

**Figure 2.**
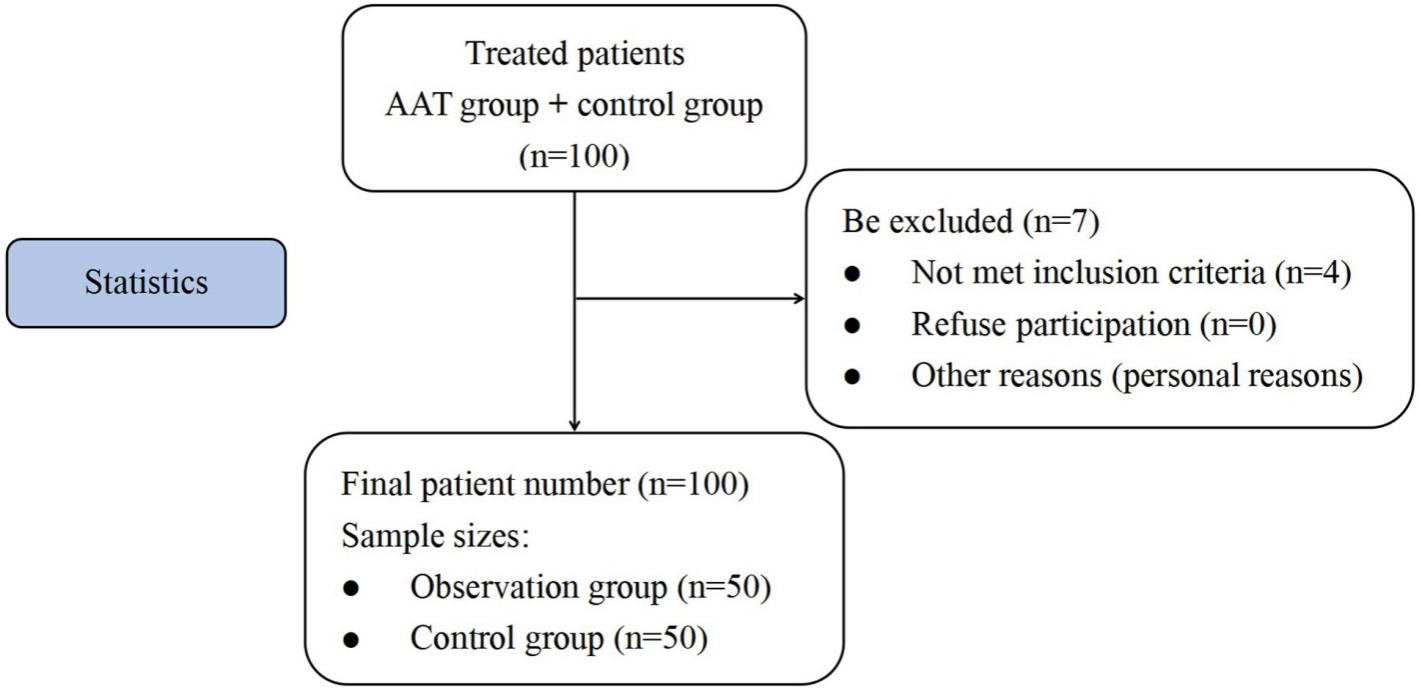
Sample size determination. Notes: The map summarizes the number of the retrospectively selected patients, patients excluded, and the final sample size obtained. n= number of samples; Control group: routine rehabilitation treatment, Observation group: auricular point bean-pressing therapy on the basis of the treatment of the routine group.

Of the 107 patients sampled in this study, 7 did not meet the inclusion criteria, and 3 were excluded based on the above clinical criteria. None of the patients refused to provide informed consent or undergo continued treatment. Follow-up was discontinued for 4 patients for personal reasons (Fig. 2). Of the 100 patients with KOA who were finally included, 50 received auricular plaster and 50 did not. None of the patients in the statistical analysis were found to have experienced complications. Among the 100 patients, 56 (56%) were women, 44 (44%) were men, 35 (35%) had Class-II KL, and 65 patients (65%) had Class-III KL. The differences in gender, age, body mass index, and KOA classification (KL type) between the two groups were not statistically significant (P> 0.05); hence, the two groups were comparable (Table 1).

### 3.2 Comparison of the knee sign scores and C-reactive protein levels between the two groups before and after treatment

**Table 2:** Comparison of swelling, tenderness, range of motion, and CRP between the two groups ($\bar{x}$± SD)

		Treatment group	Control group	P value
SQ swelling	Before treatment	2.92 ± 0.27	2.90 ± 0.30	0.730
	After treatment	1.42 ± 0.76${}^{\text{a}}$	2.64 ± 0.53${}^{\text{a}}$	<0.001
Tenderness	Before treatment	1.94 ± 0.24	1.96 ± 0.20	0.650
	After treatment	0.80 ± 0.53${}^{\text{a}}$	1.24 ± 0.66${}^{\text{a}}$	<0.001
ROM	Before treatment	2.90 ± 0.30	2.84 ± 0.37	0.377
	After treatment	1.06 ± 0.74${}^{\text{a}}$	2.40 ± 0.70${}^{\text{a}}$	<0.001
CRP	Before treatment	18.41 ± 2.15	17.82 ± 2.86	0.243
	After treatment	4.94 ± 1.42${}^{\text{a}}$	10.65 ± 2.93${}^{\text{a}}$	<0.001

Compared with the same group before treatment, Pa< 0.05.

**Table 3:** Comparison of WOMAC scores between the two groups ($\bar{x}$± SD)

		Treatment group	Control group	P value
Function	Before treatment	46.58 ± 4.56	45.48 ± 4.03	0.204
	After treatment	18.52 ± 6.12${}^{\text{a}}$	24.78 ± 4.20${}^{\text{a}}$	<0.001
Pain	Before treatment	19.48 ± 1.01	19.36 ± 1.27	0.604
	After treatment	7.40 ± 2.66${}^{\text{a}}$	14.14 ± 3.51${}^{\text{a}}$	<0.001
Stiffness	Before treatment	5.24 ± 1.41	5.16 ± 1.08	0.750
	After treatment	2.20 ± 0.93${}^{\text{a}}$	3.70 ± 1.27${}^{\text{a}}$	<0.001
Total score	Before treatment	71.30 ± 5.10	70.00 ± 4.43	0.181
	After treatment	28.12 ± 7.11${}^{\text{a}}$	42.62 ± 5.95${}^{\text{a}}$	<0.001

Compared with the same group before treatment, Pa< 0.05.

After 4 weeks of treatment, the swelling, pain, mobility score, and CRP of the knee joints in the treatment group were significantly lower than those in the control group (all P< 0.05), indicating significantly better efficacy in the treatment group (Table 2).

### 3.3 Comparison of the Western Ontario and McMaster Universities osteoarthritis scores between the two groups before and after treatment

The comparison of the total WOMAC scores between the two groups before treatment was not statistically significant (P> 0.05). After 4 weeks of treatment, the total WOMAC score of the treatment group was significantly lower than that of the control group (P< 0.05), further indicating significantly better efficacy in the treatment group (Table 3).

**Figure 3.**
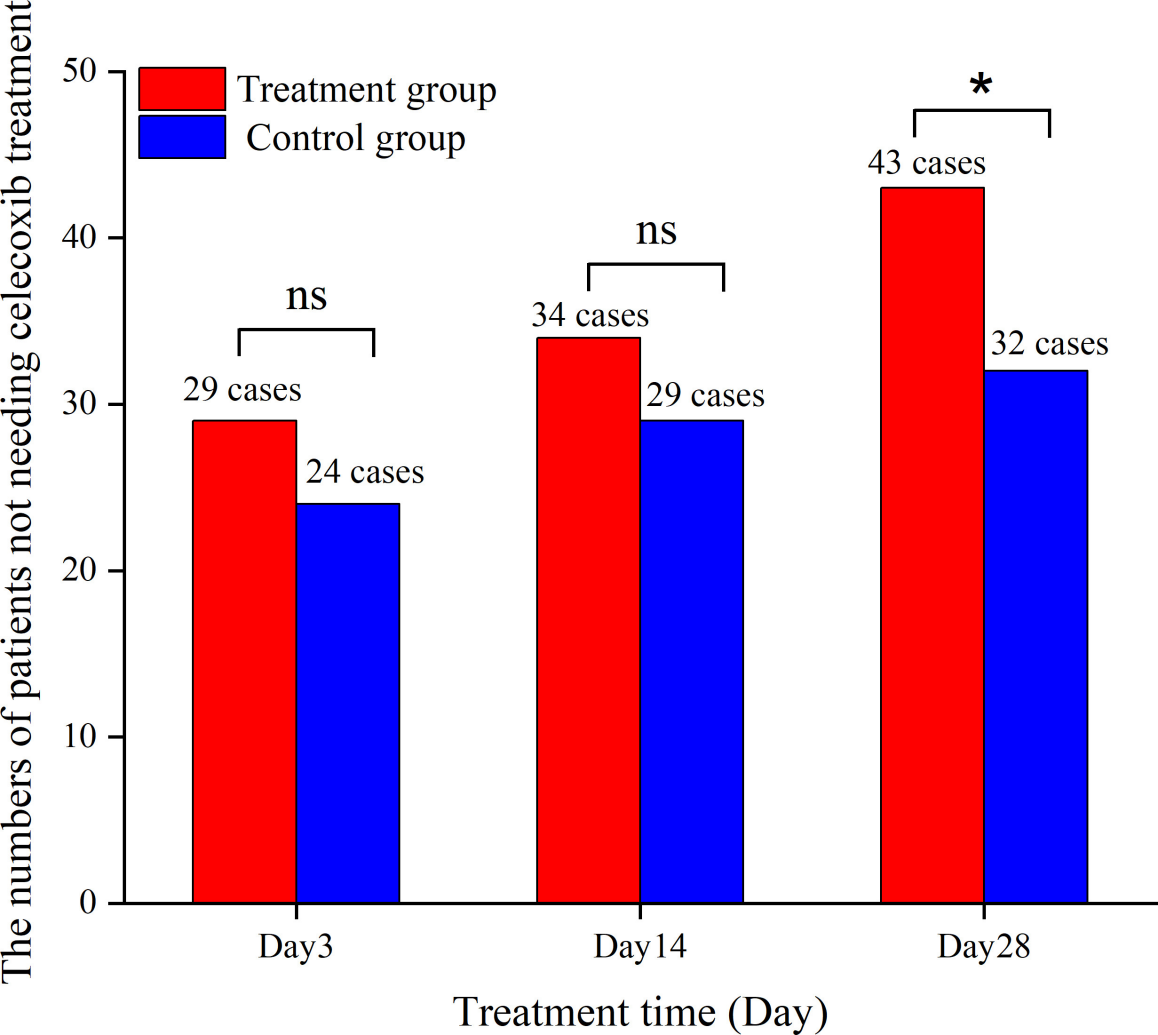
The numbers of patients not requiring celecoxib treatment in both treatment group and control group. $\chi^{2}$ test was applied for the statistical analysis of the numbers of patients in both groups. P*< 0.05 represents a statistical difference of the patients’ numbers between treatment group and control group, while ‘ns’ represents no significance between the two groups.

After 4 weeks of treatment, the NSAID requirement of the treatment group was significantly lower than that of the control group (Fig. 3).

## 4. Discussion

In the present study, the VAS and WOMAC scores of the treatment group were significantly lower than those of the control group, demonstrating that auricular bean-pressing therapy possessed analgesic and discomfort relief effects, for example, relieving mild- to moderate-degree KOA swelling, joint stiffness, and other symptoms. Considerable evidence has demonstrated the analgesic effect of auricular point bean-pressing therapy [14]. A modern study suggests that the ear is rich in nerves and blood vessels, and stimulating ear acupoints can relieve pain by increasing nerve pain thresholds and regulating the metabolism of pain-causing substances in the body [15].

The pathogenesis of KOA, a degenerative joint disease, remains unclear. It may be related to age, obesity, genetics, and other factors and can be secondary to joint injuries or joint diseases, such as meniscus rupture, joint deformity, and periarticular fractures [16]. The main pathological features of KOA are the degeneration and destruction of articular cartilage, subchondral bone sclerosis, and the formation of osteophytes at the joint edge [17]. The main causes of cartilage destruction are synovial hyperplasia, fibrosis changes, the release of cytokines, and inflammatory factors activating chondrocyte matrix metalloenzymes [18] and degrading extracellular matrix, which chondrocytes depend on for survival [19]. Another study revealed that KOA may also be related to intraosseous hypertension [20]. Here, intraosseous blood circulation in the knee joint is impaired, and intraosseous arterial perfusion is reduced, resulting in insufficient oxygen supply, which damages the nutrient and blood supply environment of the joints and ultimately accelerates the degeneration of articular cartilage. Therefore, inhibiting inflammatory response, improving intraosseous circulation, and delaying the degeneration of articular cartilage are key to preventing and treating KOA [21].

Of note, the results of the present study showed that after 28 days of intervention, the number of people in the treatment group who needed to take celecoxib was significantly lower than that of the control group, and the difference was statistically significant. The dependence on NSAIDs and related complications was also effectively improved after auricular bean-pressing therapy.

A slow, progressive, and chronic disease, osteoarthritis is the leading cause of disability among elderly people. Existing studies showed that many cytokines, growth factors [22], and signaling pathways are involved in regulating osteoarthritis [23, 24]. While NSAIDs can reduce the pain brought on by KOA, their long-term use may cause adverse effects. Auricular point bean-pressing therapy is a method based on the theory of acupuncture and moxibustion in TCM; it regulates the qi and blood of the internal organs by stimulating the acupuncture points on the ear to treat a range of diseases [25, 26]. The effect can be remarkable, particularly for patients with existing pain.

Auricular point bean-pressing therapy has the potential to treat KOA [27]. In the present study, four auricular points on the side where KOA was present, corresponding to the knee and subcortical, adrenal, and endocrinal functioning received acupoint massage. The knee acupoints corresponded to pressure points that relieved knee pain, while the adrenal, subcortical, and endocrinal acupoints were linked to sedative and analgesic effects [24]. The knee and subcortical acupoints present in the ear are holographic targets for delivering therapy to the human body; when stimulated, the feedback information derived from these points is delivered immediately to the location of the disease to mobilize the meridians and stimulate the analgesic structures in the brain to release endorphins and enkephalin analgesic substances [28, 29]. Additionally, these stimulation feedback signals can antagonize pulses emanating from the pain site to reduce pain [30]. Auricular adrenal and endocrinal acupoints can dredge meridians, regulate viscera, relieve spasm and pain, balance yin and yang, and regulate the excitation and inhibition of the cerebral cortex and disorder in the autonomic nervous center; they can also relieve cerebral cortex tension and stimulate the body to secrete pain-relieving substances, thereby inhibiting pain [31]. Tong [32] showed that the auricular adrenal and endocrinal acupoints, which were also selected in the current study, could regulate inflammatory factors and treat inflammatory lesions. These two acupoints play key roles in inhibiting the inflammatory response of the knee joint, improving intraosseous circulation, and reducing knee joint pain. Additionally, Zhang et al. [33] demonstrated that auricular acupressure on the acupoints, such as the knee joint and subcortex, plays a part in relieving analgesia and effectively decreasing NSAID requirements without inducing adverse effects when treating KOA.

Cowherb seed is a well-known TCM herb and is listed in the *Chinese Pharmacopoeia*, which is widely used in auricular point therapy in China. The seeds have no intrinsic therapeutic value but simply provide physical stimulation to the acupoints and are thus suitable for clinical auricular point bean-pressing therapy.

The small sample size of the present single-center study may have led to some error in the experimental results. Additionally, the short follow-up period in the present study made it difficult to determine the long-term effects of auricular bean pressing on patients with KOA. Further research should be undertaken without these limitations.

## 5. Conclusion

The present study demonstrated that compared with conventional rehabilitation, auricular point bean-pressing therapy combined with conventional rehabilitation treatment can further relieve knee pain, improve patients’ knee function, and reduce the demand for NSAIDs. This reduces adverse reactions caused by taking drugs, indicating that auricular point bean-pressing therapy combined with conventional rehabilitation treatment could be an effective strategy in treating KOA pain. 

## Ethical approval

The study was conducted in accordance with the Declaration of Helsinki (as was revised in 2013). The study was approved by the ethics committee of the Wenzhou Hospital of Traditional Chinese Medicine Affiliated to Zhejiang University of Traditional Chinese Medicine (approval no. WTCM-H-KT-2019047).

## Funding

The study was supported by Wenzhou Basic Scientific Research Projects (No. Y20190732).

## Informed consent

All patients provided signed informed consent for their inclusion in the research.

## Author contributions

Conception and design of the research: Yeyan Lin, Xuelai Zhou.

Acquisition of data: Yeyan Lin, Bin Shen.

Analysis and interpretation of the data: Yeyan Lin, Cunxian Lv.

Statistical analysis: Yeyan Lin, Yongqin Wu.

Obtaining financing: Xuelai Zhou.

Writing of the manuscript: Yeyan Lin.

Critical revision of the manuscript for intellectual content: Yeyan Lin.

All authors read and approved the final draft.
